# A Novel Approach Using FDG-PET/CT-Based Radiomics to Assess Tumor Immune Phenotypes in Patients With Non-Small Cell Lung Cancer

**DOI:** 10.3389/fonc.2021.769272

**Published:** 2021-11-17

**Authors:** Jianyuan Zhou, Sijuan Zou, Dong Kuang, Jianhua Yan, Jun Zhao, Xiaohua Zhu

**Affiliations:** ^1^ Department of Nuclear Medicine and PET, Tongji Hospital, Tongji Medical College, Huazhong University of Science and Technology, Wuhan, China; ^2^ Department of Pathology, Tongji Hospital, Tongji Medical College, Huazhong University of Science and Technology, Wuhan, China; ^3^ Shanghai Key Laboratory of Molecular Imaging, Shanghai University of Medicine and Health Sciences, Shanghai, China; ^4^ School of Basic Medicine, Tongji Medical College, Huazhong University of Science and Technology, Wuhan, China

**Keywords:** radiomics, tumor microenvironment immune types, non-small cell lung cancer, ^18^F-FDG PET/CT, PD-L1

## Abstract

**Purpose:**

Tumor microenvironment immune types (TMITs) are closely related to the efficacy of immunotherapy. We aimed to assess the predictive ability of ^18^F-fluorodeoxyglucose positron emission tomography/computed tomography (^18^F-FDG PET/CT)-based radiomics of TMITs in treatment-naive patients with non-small cell lung cancer (NSCLC).

**Methods:**

A retrospective analysis was performed in 103 patients with NSCLC who underwent ^18^F-FDG PET/CT scans. The patients were randomly assigned into a training set (n = 71) and a validation set (n = 32). Tumor specimens were analyzed by immunohistochemistry for the expression of programmed death-ligand 1 (PD-L1), programmed death-1 (PD-1), and CD8+ tumor-infiltrating lymphocytes (TILs) and categorized into four TMITs according to their expression of PD-L1 and CD8+ TILs. LIFEx package was used to extract radiomic features. The optimal features were selected using the least absolute shrinkage and selection operator (LASSO) algorithm, and a radiomics signature score (rad-score) was developed. We constructed a combined model based on the clinical variables and radiomics signature and compared the predictive performance of models using receiver operating characteristic (ROC) curves.

**Results:**

Four radiomic features (GLRLM_LRHGE, GLZLM_SZE, SUVmax, NGLDM_Contrast) were selected to build the rad-score. The rad-score showed a significant ability to discriminate between TMITs in both sets (*p* < 0.001, *p* < 0.019), with an area under the ROC curve (AUC) of 0.800 [95% CI (0.688–0.885)] in the training set and that of 0.794 [95% CI (0.615–0.916)] in the validation set, while the AUC values of clinical variables were 0.738 and 0.699, respectively. When clinical variables and radiomics signature were combined, the complex model showed better performance in predicting TMIT-I tumors, with the AUC values increased to 0.838 [95% CI (0.731–0.914)] in the training set and 0.811 [95% CI (0.634–0.927)] in the validation set.

**Conclusion:**

The FDG-PET/CT-based radiomic features showed good performance in predicting TMIT-I tumors in NSCLC, providing a promising approach for the choice of immunotherapy in a clinical setting.

## Introduction

Lung cancer is the leading cause of cancer-related deaths in the United States (1). Among the common subtypes of lung cancer, non-small cell lung cancer (NSCLC) represents approximately 85% of lung cancer cases. Most of the patients with NSCLC are already at an advanced stage upon diagnosis, whose 5-year survival rate is below 5% (2). Recently, immune checkpoint inhibitors targeting the programmed death-1 (PD-1)/programmed death-ligand 1 (PD-L1) axis have become standard treatments for patients with advanced NSCLC. Biomarkers, including the tumor proportion score of PD-L1, are being tested in clinical trials for its ability to identify patients who are most likely to benefit from immunotherapy (3). However, the predictive ability of PD-L1 expression is still under debate (4), since the majority of patients with PD-L1-positive tumors did not respond to PD-1/PD-L1 blockade. In addition to cancer cells, tumor immune microenvironment also plays a critical role in immunotherapy. Recent studies have demonstrated that tumor tissue dampened the host immune response by upregulation of PD-L1, which subsequently ligated to PD-1 on the antigen-specific CD8+ T cells (5). Therefore, without the preexistence of CD8+ tumor-infiltrating lymphocytes (TILs), blockade of PD-L1 or PD-1 is unlikely to achieve any antitumor efficacy. Tumor immune microenvironment could be classified into four types according to the status of PD-L1 expression and CD8+ TIL abundance (6, 7), while the tumors with tumor microenvironment immune type I (TMIT-I), i.e., with high PD-L1 expression and presence of CD8+ TILs, are more likely to benefit from anti-PD-L1/PD-1 therapies (6). An accurate identification of the TMIT-I subset not only can maximize the therapeutic efficacy of anti-PD-1/PD-L1 therapy but also can minimize the adverse effects of treatments. However, to date, there are no noninvasive methods to specifically identify the TMITs of NSCLC tumors.

Medical imaging allows a noninvasive evaluation of tumor and its microenvironment, as well as a longitudinal assessment of tumor progression. ^18^F-fluorodeoxyglucose positron emission tomography/computed tomography (^18^F-FDG-PET/CT) is one of the most commonly used diagnostic imaging modalities in oncology (8). ^18^F-FDG PET monitors the metabolism of glucose that is actively entrapped as nutrients in neoplastic tissues and tumor-associated activated immune cells (9). Therefore, ^18^F-FDG PET signals depicting the glucose metabolism are closely related to the characteristics of tumor immune microenvironment. Previous studies have shown a direct association between the maximum standardized uptake value (SUVmax) of ^18^F-FDG-PET and the expression of tumor-related immunity markers within the tumor immune microenvironment (10, 11). However, the SUVmax does not account for the spatial heterogeneity in the metabolism and biological features of tumor. Its predictive value on patients treated with immune checkpoint inhibitors remains weak. Tumor heterogeneity poses a significant challenge to personalized cancer medicine. The heterogeneity in the tumor uptake of FDG is of clinical importance as evidenced by a number of clinical trials (12). However, little attention is paid to the association between tumor immune microenvironment and the intratumoral heterogeneity of ^18^F-FDG uptake. Radiomics is a rapidly evolving field of research that is focused on the extraction and quantification of patterns within medical images (13). Unlike biopsies that only take a snapshot within a small tumor portion, radiomics captures heterogeneity across the entire tumor volume.

This retrospective study was conducted to establish a correlation between the intratumoral heterogeneity of ^18^F-FDG PET signals and tumor immune phenotype in a cohort of treatment-naive NSCLC patients. We hypothesized that radiomic features would provide insights into TMIT categorization and help optimize patient selection for immunotherapy.

## Materials and Methods

### Patients

With approval from the institutional review board, we retrospectively analyzed consecutive patients who had been diagnosed pathologically with NSCLC between December 2014 and December 2017 at our institution. Enrollment eligibility: patients histologically confirmed to present NSCLC and underwent initial ^18^F-FDG PET/CT scan within 30 days of surgery or biopsy; tumor size ≥1 cm in diameter. Exclusion criteria: patients who received antitumor therapy before surgery or biopsy due to the concern of therapy-induced alteration in PD-L1 expression. Patients without available tumor specimens for immunohistochemistry were also excluded.

### Immunohistochemistry Analysis

Immunohistochemistry was performed using protocols described in a previous study (14). In brief, 4-µm continuous sections were prepared from formalin-fixed, paraffin-embedded (FFPE) tissue blocks. Slides were autostained by the Leica Bond-Max automation (www.leica-microsystems.com) with primary antibodies against CD8 (ZA-0508, ZSGB-BIO), PD-1 [Abcam, EPR4877(2), ab137132], and PD-L1 (ZA-0629, ZSGB-BIO). The analysis of Immunohistochemistry results was performed as our previous study (15). The PD-L1 immunostaining results were classified into two groups based on staining intensity and proportion of tumor cell positivity. Staining intensity was scored as 0-3: 0, negative staining; 1, weak staining; 2, moderate staining; and 3, strong staining (more intense than alveolar macrophages). Cases with more than 5% of tumor cells and staining intensity ≥2 were defined as positive. Cases with staining intensity <2 or with positive staining in less than 5% of tumor cells were defined as negative. The expressions of PD-1 and CD8+ TILs were evaluated according to the average number of positively stained cells in three randomly selected high-power fields (HPFs) in each case. The numbers of CD8+ TILs were classified into two groups based on the median value (n=99): CD8+ TILs- (n ≤ 99) and CD8+ TILs+ (n > 99).

Four TMITs were classified as reported (6): TMIT-I (PD-L1+, CD8+ TILs+), TMIT-II (PD-L1-, CD8+ TILs-), TMIT-III (PD-L1+, CD8+ TILs-), and TMIT-IV (PD-L1-, CD8+ TILs+).

### ^18^F-FDG PET/CT Acquisition Protocol and Image Analysis

^18^F-FDG was intravenously administered at a dose of 3.7 MBq/kg after fasting for at least 6 h. The blood glucose concentration was lower than 11 mmol/L before injecting ^18^F-FDG. PET/CT imaging was performed on a PET/CT scanner (Discovery 690 PET/CT, GE) at 60 ± 5 min after FDG administration. Whole-body images were obtained from the base of the skull to mid-thigh by means of an integrated PET/CT tomography (5–7 bed positions with 2 min per bed position). A low-dose helical CT scan (120 kV; 120 mA; slice thickness, 3.75 mm) was performed for anatomical correlation and attenuation correction. Reconstructed images were then displayed on a GE ADW4.5 workstation. Tumor mass was identified as the volume with elevated ^18^F-FDG uptake compared to normal lung parenchyma or other mediastinal structures. SUVmax was defined as the highest pixel value of PET imaging. Tumor burden was calculated by drawing a three-dimensional volume of interest (VOI) on the volume of tumor-related metabolic activity and applying a percentage threshold at 30% of SUVmax.

### Radiomic Feature Extraction

The feature extraction was performed as previously described (16). Briefly, LIFEx package (version 5.10, http://www.lifexsoft.org) was used to extract the texture features of ^18^F-FDG PET/CT images of lesions in the same VOI. The ^18^F-FDG PET/CT images of the patient in the DICOM format were imported into the software. Two experienced PET/CT diagnostic physicians semiautomatically delineated the VOI of the target lesion using a threshold at 30% of SUVmax. The interobserver reliability between the two physicians was analyzed. Then, the software program automatically calculates and extracts 52 PET radiomic features and 51 CT radiomic features, which are provided in **Supplemental Table 1**.

### Radiomic Feature Selection and Model Establishment

Radiomic features with significant differences among different TMITs were selected in the training set using the Mann–Whitney U test with a *p*-value <0.05. The least absolute shrinkage and selection operator (LASSO) algorithm with 10-fold cross-validation was then used to select the optimal predictive features in the training set. The selected features with non-zero coefficients at the minimum of lambda were selected to construct a radiomics signature score (rad-score). Finally, rad-score and clinical variables were combined to establish a complex model using multivariate logistic regression analysis.

Model performance was tested in the validation set. Briefly, receiver operating characteristic (ROC) curve and area under the ROC curve (AUC) were used to evaluate the model performance in the training and validation sets. A nomogram was developed to display the prediction results for each patient using the rad-score and clinical variables, and calibration curves were plotted to improve the nomogram’s prediction accuracy. Furthermore, decision curve analysis (DCA) was performed to evaluate the clinical usefulness of the combined model by quantifying the net benefits at different threshold probabilities.

### Statistical Analysis

All statistical tests were performed using SPSS statistical package (version 22.0, IBM, Armonk, NY, USA), MedCalc (MedCalc Software bvba, Ostend, West Flanders, Belgium), and R version 3.6.2 (http://www.r-project.org).

Feature reliability was analyzed using an intraclass correlation coefficient (ICC), where ICC ≥0.75 is generally considered to indicate good repeatability of the measured results. Mann–Whitney U test and Fisher’s exact test were used to test the differences between continuous variables or categorical variables, respectively. Relations between two variable distributions were analyzed with the Spearman rank correlation coefficient (rho).

R package “glmnet” was used to perform LASSO binary logistic regression analysis, “rms” package to create the nomogram and calibration curve, “rmda” package to plot the DCA, “ggplot” package to plot the bar graph, and the “pROC” package to analyze ROC curves. A *p*-value <0.05 was considered statistically significant.

## Results

### Patient Characteristics

In total, 103 patients were eligible for the retrospective analysis. The median age of the patients was 59 years old (range: 33–78 years old). The patients were randomly assigned to training or validation set at a ratio of 7 to 3, with 71 cases in the training set and 32 in the validation set. The baseline characteristics of the patients are summarized in **Table 1**.

**Table 1:** Demographic and clinical data of all patients.

Variables		All patients (n = 103)	Training set (n = 71)	Validation set (n = 32)	*p*
Age (years)	Range	59 (33–78)	56 (33–78)	63 (49–76)	0.03
Gender	Male	57	35	22	0.09
	Female	46	36	10	
Smoking	Smoker	45	26	19	0.03
	Non-smoker	58	45	13	
Histology	SCC	28	17	11	0.208
	No-SCC	75	54	21	
Stage	I	37	23	14	0.20
	II	24	16	8	
	III	30	23	7	
	IV	12	9	3	
SUVmax	Range	9.49 (0.88–23.5)	9.61 (0.88–23.5)	9.00 (1.19–21.0)	0.932

SCC, squamous cell carcinoma.

### Feature Reliability

Feature extraction was performed by two physicians to ensure the validity and reproducibility of the procedure. After examining the inter-set differences with Manny–Whitney U-test, as well as the interobserver reliability with ICC, it was concluded that none of the features was significantly different from each other (*p* > 0.05), suggesting that all the features were reliable and reproducible (ICC > 0.75).

### Correlations Between Radiomic Features and Immune Variables

By Mann–Whitney U test, 51 radiomic features were significantly different between PD-L1+ and PD-L1- patients (*p* < 0.05) (**Supplemental Table 2**). ROC for these indices showed moderate ability for predicting PD-L1 expression (AUC < 0.710), and the preferable features in differentiating PD-L1 status include SUVmax (AUC = 0.704) among the basic features and GLRLM_LRHGE (AUC = 0.702) and GLRLM_HGRE (AUC = 0.700) among the texture features.

Thirty-seven radiomic features correlated with CD8+ TILs in NSCLC (rho = -0.289 to 0.310, *p* < 0.05), among which NGLDM_Contrast has a strong correlation with CD8+ TILs with the largest linear correlation coefficient (rho = 0.310, *p* = 0.001; **Supplemental Table 3**).

In addition, PD-1+ TILs correlated with abundant radiomics indices, including 40 PET features and 28 CT features (rho = -0.317 to 0.356, *p* < 0.05; **Supplemental Table 3**). The strongest correlation was between SUVpeak (1 ml) and PD-1 expression (rho = 0.356, *p* < 0.001; **Supplemental Table 4**).

### Feature Extraction

**Figure 1:**
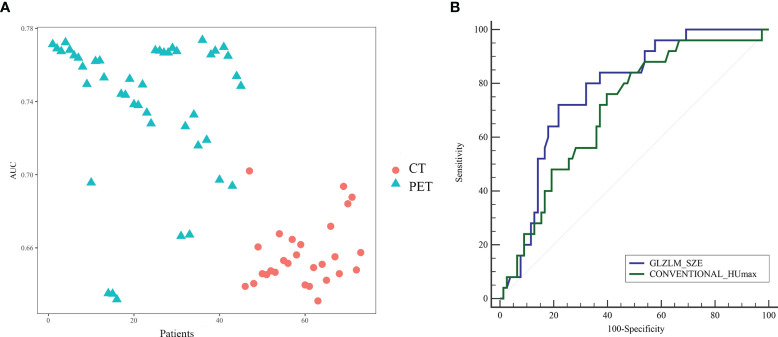
**(A)** The performance of radiomic features for the evaluation of tumor microenvironment immune type (TMIT)-I tumors. All the features showed moderate power for predicting the TMIT-I tumors, and PET features have better ability than CT features with higher area under the receiver operating characteristic (ROC) curve (AUC). Panel **(B)** was the ROC curve of the optimal PET (GLZLM_SZE) and CT (Conventional_HUmax) features to distinguish TMIT-I from other groups.

To avoid model overfitting, radiomic features with *p*-values <0.05 were first selected by the Mann–Whitney U test. Seventy-three features (42 PET features, 31 CT features) were found significantly different among TMIT groups in the training set. All of these features showed moderate power for predicting the TMIT-I tumors (**Figure 1**).

### Construction of the Radiomics Signature and Complex Model

**Figure 2:**
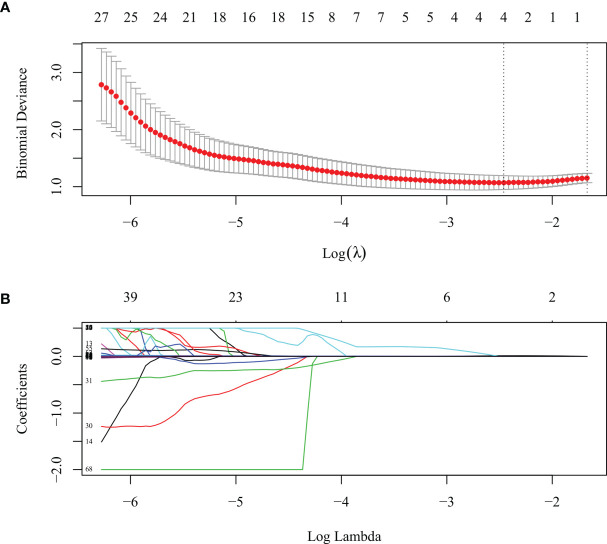
The least absolute shrinkage and selection operator (LASSO) algorithm and 10-fold cross-validation were used to extract the optimal subset of radiomic features. **(A)** Tuning parameter (lambda, λ) selection in the LASSO model used 10-fold cross validation for the training set. The mean deviance (goodness-of-fit statistics, red dots) was plotted vs. log (λ), error bars displaying the range of standard error. Dotted vertical lines were drawn at the point of minimum deviance and at the point where maximum λ was obtained among errors smaller than the standard error of minimum deviance. **(B)** LASSO coefficient profiles of the 73 texture features.

LASSO algorithm and 10-fold cross-validation were used to extract the optimal subset of radiomic features from the 73 features above. Four radiomic features were then selected to build the radiomics signature score based on the 71 patients in the training set (**Figure 2**) as follows: GLRLM_LRHGE, GLZLM_SZE, SUVmax, and NGLDM_Contrast. The first three were PET features, and the last one was a CT feature. A rad-score for each patient was calculated using the following formula:


(1) $$\begin{array}{c} Rad-score=GLZLM\_SZE\times 0.6929504962+GLRLM\_LRHGE\times 0.0001966283+SUV\max \end{array}$$

**Figure 3:**
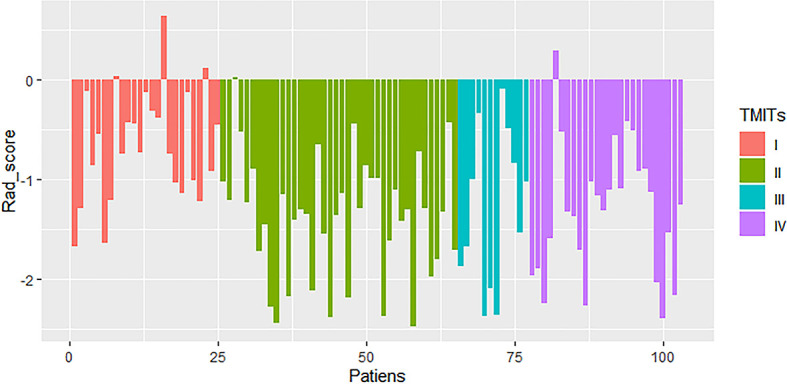
Rad-score of patients in the cohort of patients with non-small cell lung cancer (NSCLC). Generally, rad-scores in the tumor microenvironment immune type (TMIT)-I tumors were higher than other TMIT-II~IV.

The median and the range for the four selected radiomic features and the calculated rad-score are shown in **Table 2**. The rad-score and the four selected features were significantly different among the TMITs in both the training and the validation sets (*p* < 0.05). The rad-score for each patient in the two sets was displayed as a bar graph in **Figure 3**.

**Table 2:** The differences of four selected radiomic features and the calculated rad-score between TMITs.

Variables	Training set (n = 71)		*p*	Validation set (n = 32)		*p*
	TMIT-I (n = 18)	TMIT-II~IV (n = 53)		TMIT-I (n = 7)	TMIT-II~IV (n = 25)	
Rad-Score	-0.645 (-1.67 to 0.64)	-1.328 (-2.47 to -0.08)	0.000157	-0.734 (-1.223 to 0.123)	-1.286 (-2.39 to 0.289)	0.018895
***PET features***						
SUVmax	12.65 (5.10–23.50)	8.03 (0.875–19.50)	0.000634	13.70 (8.01–19.40)	8.13 (1.19–21.00)	0.024031
GLRLM_LRHGE	617 (85.90–2,680)	242 (18.50–1,440)	0.000750	664.0 (254–1,180)	276 (25.8–1770)	0.030368
GLZLM_SZE	0.697 (0.514–0.797)	0.548 (0.001–0.872)	0.000438	0.707 (0.421–0.763)	0.535 (0.017–0.791)	0.040220
***CT feature***						
NGLDM_Contrast	37.40 (0–72.80)	15.10 (0–62.60)	0.009743	30.7 (14.6–61.6)	21.30 (0–78.4)	0.171421

TMIT, tumor microenvironment immune type.

With multivariate logistic regression analysis (using backward stepwise elimination method), the combined model was constructed based on the clinical variables (age, gender, smoking history, stage) and radiomics signature. The formula was as follows:


(2) Model−score=1.668×rad−score+1.481×smoking−0.121

### Performance of the Radiomics Signature and Clinical Features

**Figure 4:**
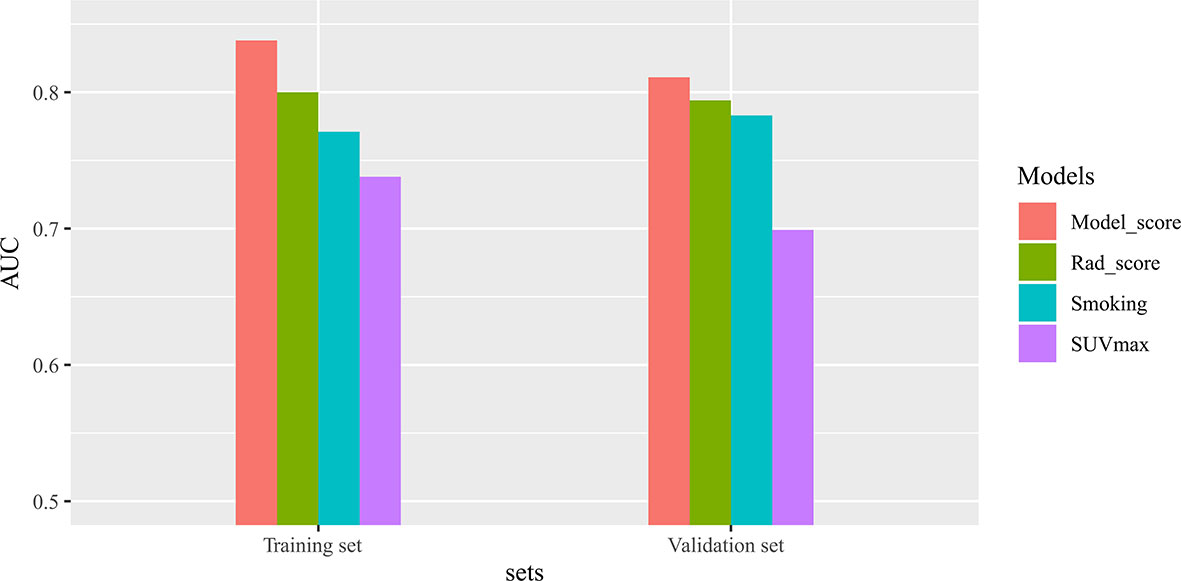
The differences of area under the receiver operating characteristic (ROC) curve (AUC) values in different variables. There are significant differences in AUCs between Model-score and smoking in both the training and validation sets.

We evaluated the models based on radiomics signature, clinical variables (smoking history), and the complex model in terms of their ability to predict TMIT-I tumors. The complex model had good predictive ability, and its AUCs in differentiating TMIT groups were 0.838 [95% CI (0.731–0.914)] in the training set and 0.811 [95% CI (0.634–0.927)] in the validation set. The predictive abilities of the four models, including sensitivity and specificity, were shown in **Table 3**. The differences of AUC values in different variables were shown in **Figure 4**. Notably, the AUCs of the complex model and smoking history were significantly different in the training set and validation set (*p* = 0.0156, *p* = 0.0250). The AUC values of the complex model and radiomics signature were not significantly different in either the training set or the validation set (*p* > 0.05).

**Table 3:** Predictive performance of variables in the training and validation sets.

Variables	Training set			Validation set		
	AUC (95% CI)	Sensitivity (%)	Specificity (%)	AUC (95% CI)	Sensitivity (%)	Specificity (%)
Model-score	0.838 (0.731–0.914)	72.22%	88.68%	0.811 (0.634–0.927)	85.71%	76.00%
Rad-Score	0.800 (0.688–0.885)	66.67%	81.13%	0.794 (0.615–0.916)	100%	56.00%
Smoking	0.738 (0.621–0.836)	72.22%	75.47%	0.699 (0.481–0.824)	85.71%	48.00%
SUVmax	0.771 (0.656–0.862)	72.22%	73.58%	0.783 (0.602–0.908)	85.71%	72.00%

### Individualized Nomogram Construction and Validation

**Figure 5:**
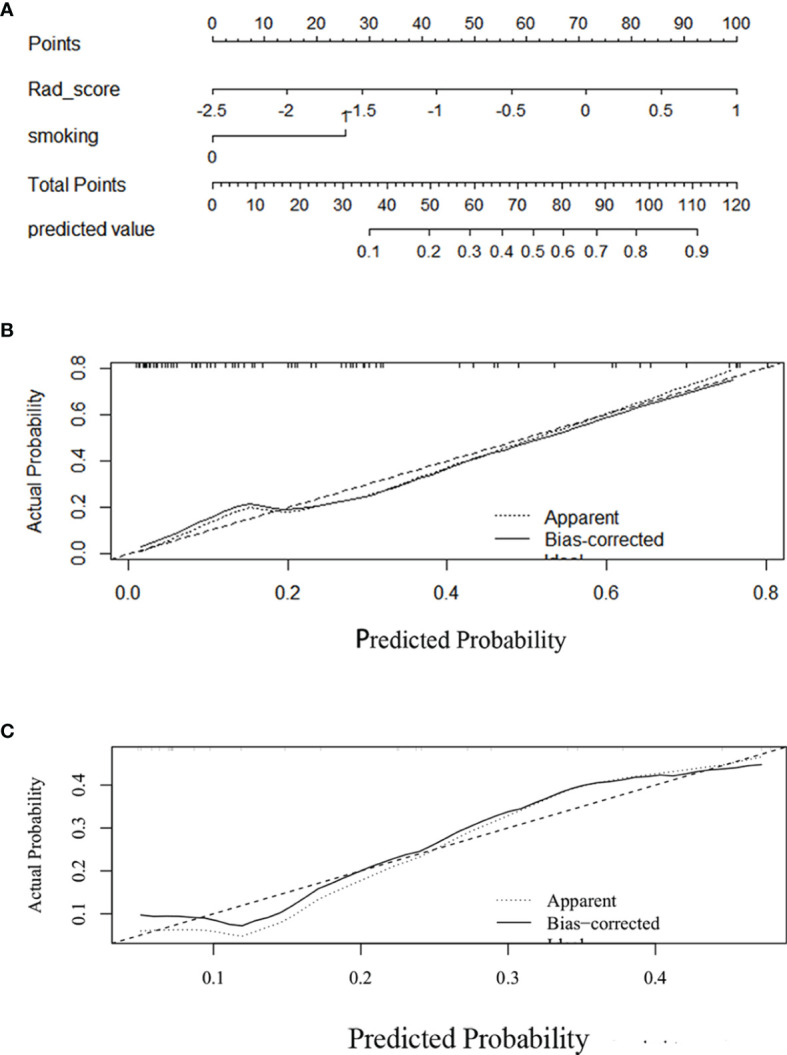
Development and performance of a nomogram. **(A)** Nomogram based on rad-score and clinical factors (smoking history). Calibration curves **(B, C)** of the nomogram in the training set. The horizontal axis is the predicted incidence of the tumor microenvironment immune type (TMIT)-I tumors. The vertical axis is the observed incidence of the TMIT-I tumors. The diagonal line is the reference line, indicating that the predicted value is equal to the actual value. The prediction results and diagonals were basically coincident, indicating that the prediction results were accurate.

**Figure 6:**
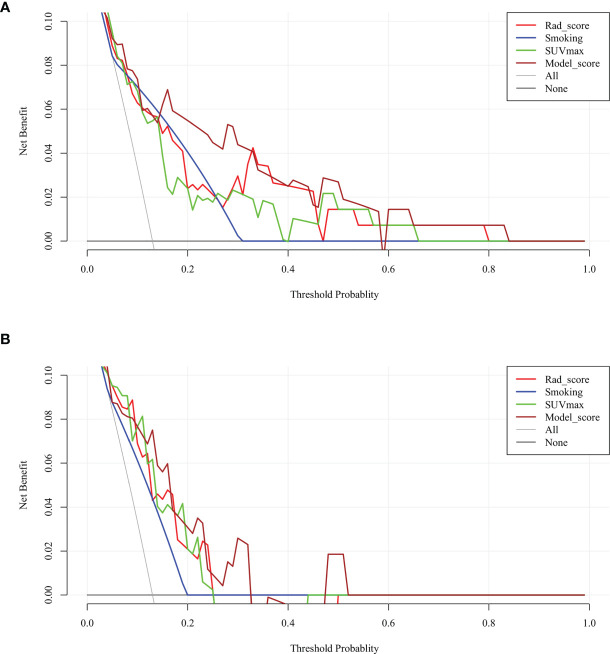
Decision curve analysis (DCA) of each model in predicting tumor microenvironment immune type (TMIT)-I for non-small cell lung cancer (NSCLC). The vertical axis measures standardized net benefit. The horizontal axis shows the corresponding risk threshold. In the training set **(A)**, the DCA showed that if the threshold probability is between 0.1 and 0.8, using the complex model (brown line) provided a greater benefit than the clinical model (blue curve) and basic PET parameter (green curve). In the validation set **(B)**, the DCA showed that if the threshold probability is between 0.1 and 0.5, using the complex model provided a greater benefit than the clinical model.

**Figure 7:**
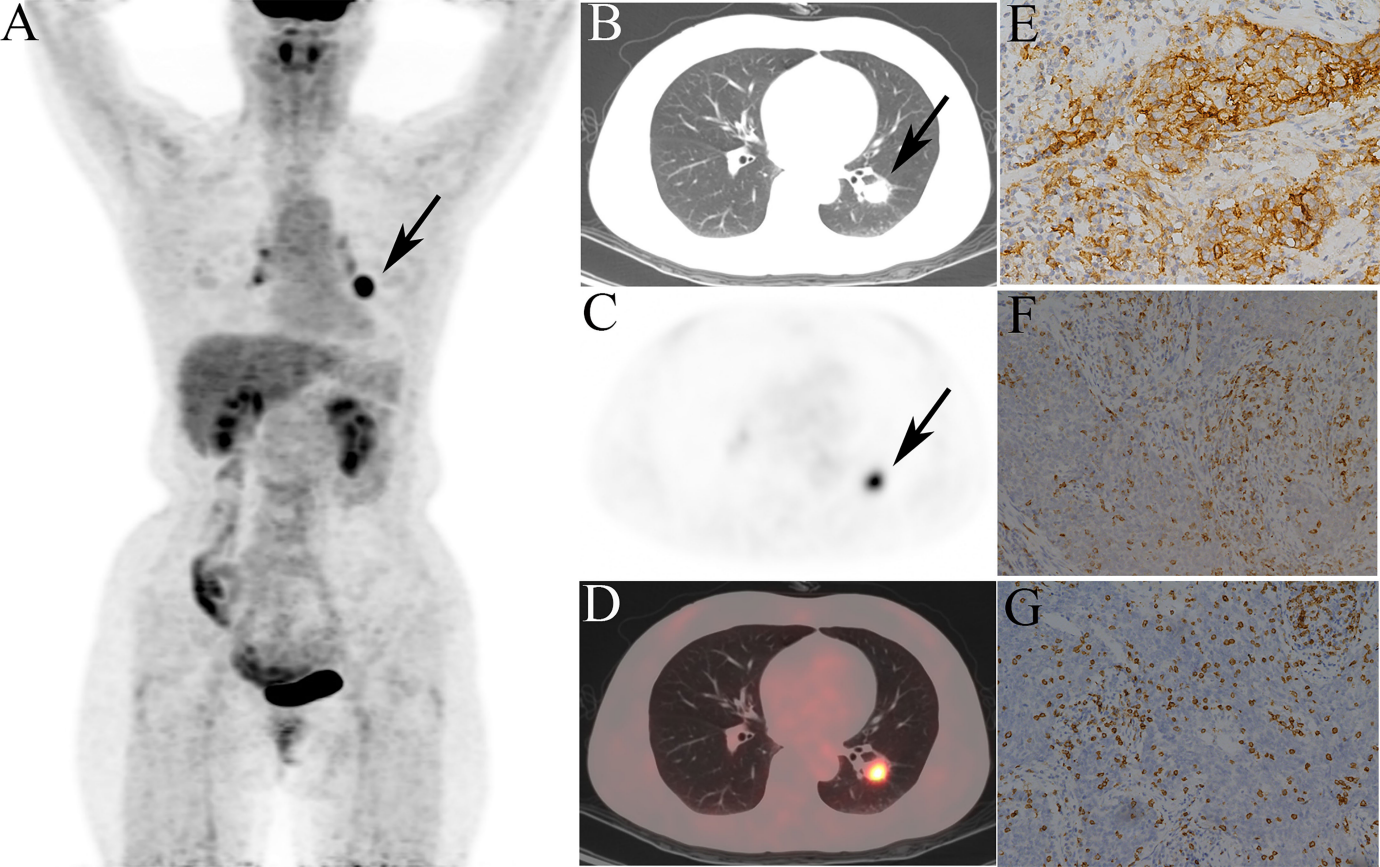
Representative ^18^F-fluorodeoxyglucose positron emission tomography/computed tomography (^18^F-FDG PET/CT) images of a 52-year-old female patient, defined as tumor microenvironment immune type (TMIT)-I tumor. **(A)** MIP figure, **(B–D)** CT, PET, PET/CT fusion images. A nodule was in the lower lobe of the left lung (arrow) with markedly increased radioactivity, SUVmax: 10.3, GLRLM_LRHGE: 382, GLZLM_SZE: 0.682, and GLCM_Contrast: 14.6. The surgical pathology: poorly moderately differentiated squamous cell carcinoma. **(E)** High programmed death-ligand 1 (PD-L1) expression. **(F)** Programmed death-1 (PD-1) tumor-infiltrating lymphocytes (TILs) high density. **(G)** CD8+ TILs high density.

Given that the complex model based on both rad-score and clinical variables had better ability to predict TMIT-I tumors, we created a nomogram representing the individualized predictions based on the training set, which visualized the prediction result and the proportion of each factor (**Figure 5A**) . The calibration curves of the nomogram in the training and validation sets were presented in **Figures 5B, C** and showed good agreement between the predicted and observed values in the training set. DCA for the combined model (**Figure 6**) showed that prediction of TMIT-I tumors with the complex model added more benefit to SUVmax and the clinical variable (smoking history) in the training set. **Figure 7** showed that a representative patient with a TMIT-I type exhibited a hypermetabolic and heterogeneous tumor on ^18^F-FDG PET, characterized by high expression of PD-L1 and high density of PD-1, CD8+ TILs.

## Discussion

The past decade was marked by a revolution in the treatment of NSCLC, including the variety of immunotherapy strategies targeting the tumor immune microenvironment (17–19). Biomarkers, such as TMIT-I, can identify the patient population that are more likely to respond to the immunotherapy (6). Consequently, novel approaches to assess the tumor immune microenvironment are of particular interest in clinical practice. We strived to address this need by proposing an ^18^F-FDG-PET/CT-based radiomics to assess TMITs, especially TMIT-I tumors in pretreatment NSCLC patients. To the best of our knowledge, this is the first attempt to identify this type of immune “hot” tumors using PET/CT-based radiomics in pretreatment NSCLC patients.

Among the selected features in our work, numerous indices, including basic and texture features, were associated with PD-L1/PD-1 expression and CD8+ TILs. The metabolic characteristics of PD-L1/PD-1 expression in lung cancer were revealed in the previous study (10, 11). It seems that PD-L1-positive cells take up more glucose. Tumor microenvironment with high PD-L1 expression is often accompanied with dysfunctional anti-tumor immune responses, and therefore can foster immune tolerance that is favorable for tumor progression (20). However, the molecular mechanism between glucose metabolism and PD-L1 expression has not been fully revealed. Chang et al. (21) reported that PD-L1 expression maintained Akt/mammalian target of rapamycin (mTOR) signaling, which in turn promoted metabolic pathway through the translation of glycolysis enzymes. It might partly explain that the PD-L1/PD-1-positive tumors were more heterogeneous with more ^18^F-FDG involvement in tumor cells. Moreover, the PD-L1 protein expression has been noted to be heterogeneous within different intertumoral regions, and the distribution of expression was frequently present near stromal tumor interfaces (22). Some tumors may display heterogeneous PD-L1 expression at different biopsied sites, which may partly explain the reason of mixed response to anti-PD-L1/PD-1 therapy (23, 24). On the other hand, the distribution of PD-L1 expression may cause different metabolic distributions of tumor cells. As is well known, the heterogeneity of image voxel intensities can be quantified by different image processing and analysis methods, including texture analysis, thus texture features describe the uniformity and heterogeneity of the PET images. These metabolic patterns could be representative of the intratumoral heterogeneous expression of PD-L1/PD-1.

Previously, quantitative CT radiomic features were extracted to predict PD-L1 expression in advanced-stage lung adenocarcinoma, yet their ability to predict PD-L1 positivity was weak (AUC = 0.661) (25). Recently, radiomics models of PET/CT demonstrated good performance in classifying a group of patients with PD-L1 expression, either ≥1% or ≥50%; however, TILs were not included in their research (26). Meanwhile, Jiang et al. (26) found that the performance of PET features was still unsatisfying, although the radiomics-based signatures from CT data achieved significant and robust individualized estimation of specific PD-L1 status. In this study, we used ^18^F-FDG-PET/CT-based radiomics to analyze the correlation between radiomic features and PD-L1 expression. Among the numerous parameters, GLRLM_LRHGE derived from the PET images is the preferable feature to discriminate the PD-L1 status and achieved a moderate performance of predicting PD-L1. GLRLM reflects the comprehensive information of the image gray scale with respect to direction, adjacent interval, and variation amplitude (27), which is a set of statistical features extracted from medical images and frequently applied in radiomics (28, 29). Long-Run High Gray-level Emphasis (LRHGE) is the distribution of the long homogeneous runs with high gray levels. This may reflect that intertumoral regions with high PD-L1 expression are associated with high gray levels (or high metabolic distribution) on PET images.

Interestingly, NGLDM_Contrast had a strong correlation with CD8+TILs. NGLDM_Contrast measures the intensity difference between neighboring regions. Several studies have shown that preexisting tumoral and peritumoral immune infiltration correlates with patient response to anti-PD-1 and anti-PD-L1 therapy (30). CD8+ TILs are not evenly distributed within the tumor, where both T cell-infiltrated and T cell-excluded regions are present (31). It is likely that the heterogeneous distribution of CD8+ TILs contributed to the heterogeneity pattern of tumor metabolism, which was depicted by NGLDM_Contrast.

Rad-signature and complex model showed better predictive performances for TMIT-I tumors compared to the conventional features (SUVmax) and clinical variables probably because SUVmax alone does not accurately recapitulate the spatial heterogeneity of tumor metabolism (32). Radiomics aims to extract quantitative information from medical images that are difficult to be recognized or quantified by human eyes (33). Until recently, Sun et al. (34) developed a radiomics signature predictive of immunotherapy response by combining contrast-enhanced CT images and RNA-seq genomic data. The signature was able to discriminate inflamed tumors from immune-desert tumors, although with a modest AUC value of 0.76. Still, the ability of the radiomics signature to predict the gene expression signature of CD8 cells is unsatisfactory in the validation set (AUC = 0.67), underlying the importance of developing more and better imaging modality-based radiomics. We assessed the tumor immune microenvironment with ^18^F-FDG-PET/CT radiomics and provided a promising way to predict the tumor immune phenotype. The nomogram included the radiomics signature score and clinical variables, which visualized the prediction results and provided an easy-to-use method for individualized prediction of TMIT-I tumors. In addition, radiomics-based signature could provide predicting outcomes at the time of image acquisition, providing a real-time guidance for patient stratification and therapeutic efficacy prediction.

DCA was used to facilitate the comparison between different prediction models. The utility of risk models may be evaluated with cost-effectiveness studies in clinical practice (35). DCA focuses on net benefit, which combines the number of true positives and false positives into a single “net” number (36, 37). In the TMIT example, the “net” values were calculated by subtracting the false positives (inconsistent biopsies showing other types of TMITs from the true positives TMIT I tumors confirmed by biopsies).

As seen in **Figure 6**, the clinical usefulness of each model was evaluated using DCA method by plotting the “net” benefit of using the model to stratify patients (y axis) against the continuum of potential thresholds for the probability of TMIT-1 tumors (x axis) (38). This study developed and validated a complex model to identify NSCLC patients with Type-I TMIT. The novel approach was based on radiomic features, clinical variable, and ^18^F-FDG uptake. The ^18^F-FDG uptake accounted for intratumoral heterogeneity that correlated with underlying biological processes. The model described in our study showed good discriminative ability in both training and validation sets and exhibited higher predictive accuracy than conventional PET parameters (e.g., SUVmax). Within the range from 0.15 to 0.4 of the threshold probabilities, the model obviously showed a higher curve than that of SUVmax in **Figure 6**, indicating a much higher net benefit of our complex model than that of SUVmax. Therefore, this complex model obtained more true-positive cases of TMIT-I tumors and avoid more false-negative cases of other immune types. Considering the low probability of TMIT-I in clinical practice, it indicates that our DCA curve has a promising potential for clinical application. We agree that the DCA curve of the verification set is less optimal than that of the training set, which may require expansion of sample size and further optimization of the training model. Nevertheless, the DCA curves demonstrated advantages of complex model over radiomics, indicating clinical variable is also important.

To the best of our knowledge, there are no consensus cutoff values of PD-L1 and CD8+ TILs, even though the Food and Drug Administration (FDA) approved the cutoff of 50% tumor proportion score for first-line therapy with pembrolizumab and 1% tumor proportion score for second-line therapy with pembrolizumab/atezolizumab/bevacizumab (39). For the expression of PD-L1, we referred to a previous literature with a relatively large cohort and thus more reliable results (40). Koh et al. (39) evaluated PD-L1 immunohistochemistry based on the intensity and proportion of membranous and/or cytoplasmic staining in tumor cells. For CD8+ TILs, median or mean values were often used for classification of high or low infiltration (7, 41). Lin et al. (42) transferred continual variables like CD8+ T-cell infiltrating density and PD-1/PD-L1 mRNA expression level into categorical variables (high vs. low) with median value as cutoff point. Similarly, a recent assessment for PD-L1 was performed by Noh et al. (43), where PD-L1 expression was interpreted based on the proportion and intensity (graded as 0–3) of positive tumor cells. Besides, they utilized mean values as the cutoff threshold to categorize the CD8 TILs as “high” or “low.” Based on the above, PD-L1+ was defined as more than 5% of tumor cells with staining intensity ≥2, and median value >99 was for CD8+ TILs in our study.

Our study has some limitations. First, it was of a single-center design and the relatively small sample size may influence the predictive ability of radiomics signature. Therefore, it is necessary to carry out multicenter studies and test multicenter data to ensure better robustness of the model. Second, patients with both lung squamous cell carcinoma and adenocarcinoma were enrolled and investigated, and the predictive performance of each tumor subtype should be further validated separately in a larger cohort. Third, with the development of quantitative imaging methods along with machine learning, it provides powerful modeling tools to mine the huge amount of image data available and reveal underlying complex biological mechanisms (44). Therefore, more advanced radiomics approaches, such as machine learning and deep learning, should be established to develop a model with optimal prediction performance.

## Conclusion

In conclusion, a radiomics signature and complex model were developed and validated in patients with NSCLC. ^18^F-FDG-PET/CT radiomics may provide a noninvasive method for predicting tumor immune phenotypes, which can assist in clinical practice to identify candidates for immunotherapy.

## Data Availability Statement

The original contributions presented in the study are included in the article/**Supplementary Material**. Further inquiries can be directed to the corresponding author.

## Ethics Statement

The studies involving human participants were reviewed and approved by the Institutional Review Board of Huazhong University of Science and Technology, Tongji Medical College affiliated Tongji Hospital (TJ-IRB 20181202).

## Author Contributions

XZ, JYZ, and SZ contributed to the conception and design of the study. JYZ and DK carried out the acquisition of data. JYZ, SZ, JZ, JY, and XZ performed the data analysis. JYZ wrote the first draft of the article. XZ, SZ, DK, JZ, and JY made the comments. XZ and JZ critically reviewed and revised the article. All authors contributed to the article and approved the submitted version.

## Funding

This study was funded by the National Natural Science Foundation of China (91959119, 81873903, 81671718).

## Conflict of Interest

The authors declare that the research was conducted in the absence of any commercial or financial relationships that could be construed as a potential conflict of interest.

## Publisher’s Note

All claims expressed in this article are solely those of the authors and do not necessarily represent those of their affiliated organizations, or those of the publisher, the editors and the reviewers. Any product that may be evaluated in this article, or claim that may be made by its manufacturer, is not guaranteed or endorsed by the publisher.
