# Supplementary material for: A Novel Approach Using FDG-PET/CT-Based Radiomics to Assess Tumor Immune Phenotypes in Patients With Non-Small Cell Lung Cancer
Source: Front Oncol. 2021 Nov 17;11:769272. doi: 10.3389/fonc.2021.769272 (PMC8635743; doi:10.3389/fonc.2021.769272)
Supplement: Supplementary file 1 [file Table_1.docx]

**Supplemental Table 1.** Report on radiomic features extracted from the PET/CT images

| **Basic features** | | | |
| --- | --- | --- | --- |
| **Basic features PET** | **Basic features CT** | **Indices from Histogram** | **Indices from Shape** |
| SUV_minimum_  SUV_maximum_  SUV_mean_  SUV_standarddeviation_  SUV_peaksphere_  TLG | Minimum  Maximum  Mean  Standard deviation | HISTO_Skewness  HISTO_Kurtosis  HISTO_Entropy_log10  HISTO_Entropy_log2  HISTO_Energy | SHAPE_Sphericity  SHAPE_Compacity  SHAPE_Volume (mL)  SHAPE_Volume (voxels) |
| **Texture Features** | | | |
| **Grey Level Co-occurrence Matrix (GLCM)** | **Grey-Level Run Length Matrix (GLRLM)** | | |
| GLCM_Homogeneity  GLCM_Energy  GLCM_Contrast  GLCM_Correlation  GLCM_Entropy_log10  GLCM_Entropy_log2  GLCM_Dissimilarity | GLRLM_SRE (Short-Run Emphasis)  GLRLM_LRE (Long-Run Emphasis)  GLRLM_LGRE (Low Gray-level Run Emphasis )  GLRLM_HGRE (High Gray-level Run Emphasis)  GLRLM_SRLGE (Short-Run Low Gray-level Emphasis)  GLRLM_SRHGE (Short-Run High Gray-level Emphasis)  GLRLM_LRLGE (Long-Run Low Gray-level Emphasis)  GLRLM_LRHGE (Long-Run High Gray-level Emphasis)  GLRLM_GLNUr (Gray-Level Non-Uniformity for run)  GLRLM_RLNU (Run Length Non-Uniformity)  GLRLM_RP (Run Percentage) | | |
| **Neighborhood Grey-Level Different Matrix (NGLDM)** | **Grey-Level Zone Length Matrix (GLZLM)** | | |
| NGLDM_Coarseness  NGLDM_Contrast  NGLDM_Busyness | GLZLM_SZE (Short-Zone Emphasis)  GLZLM_LZE (Long-Zone Emphasis)  GLZLM_LGZE (Low Gray-level Zone Emphasis)  GLZLM_HGZE (High Gray-level Zone Emphasis)  GLZLM_SZLGE (Short-Zone Low Gray-level Emphasis)  GLZLM_SZHGE (Short-Zone High Gray-level Emphasis)  GLZLM_LZLGE (Long-Zone Low Gray-level Emphasis)  GLZLM_LZHGE (Long-Zone High Gray-level Emphasis)  GLZLM_GLNUz (Gray-Level Non-Uniformity for zone)  GLZLM_ZLNU (Zone Length Non-Uniformity)  GLZLM_ZP (Zone Percentage) | | |

The parameters were calculated according to the formulas described in the software manual (LIFEx v5.10 ).

**Supplemental Table 2.** Comparison of the radiomic features between PD-L1 positive and negative groups.

| **PET features** | **Variables** | ***z*** | ***p*** |
| --- | --- | --- | --- |
| **Basic features PET** | SUVmin | -3.303 | <0.001 |
|  | SUVmean | -3.365 | <0.001 |
|  | SUVstd | -3.423 | <0.001 |
|  | SUVmax | -3.430 | <0.001 |
|  | SUVQ1 | -3.334 | <0.001 |
|  | SUVQ2 | -3.289 | 0.001 |
|  | SUVQ3 | -3.330 | <0.001 |
|  | SUVpeak sphere 0.5mL | -3.066 | 0.002 |
|  | SUVpeak sphere 1mL | -3.074 | 0.002 |
|  | TLG (mL) | -2.361 | 0.018 |
| **Indices from Histogram** | HISTO_Entropy_log10 | -3.341 | <0.001 |
|  | HISTO_Entropy_log2 | -3.348 | <0.001 |
|  | HISTO_Energy | -3.190 | 0.001 |
| **Grey Level Co-occurrence Matrix (GLCM)** | GLCM_Homogeneity | -2.987 | 0.003 |
|  | GLCM_Energy | -3.210 | 0.001 |
|  | GLCM_Contrast | -3.135 | 0.002 |
|  | GLCM_Entropy_log10 | -3.207 | 0.001 |
|  | GLCM_Entropy_log2 | -3.200 | 0.001 |
|  | GLCM_Dissimilarity | -3.069 | 0.002 |
| **Grey-Level Run Length Matrix (GLRLM)** | GLRLM_SRE | -2.750 | 0.006 |
|  | GLRLM_LRE | -2.686 | 0.007 |
|  | GLRLM_LGRE | -3.330 | <0.001 |
|  | GLRLM_HGRE | -3.358 | <0.001 |
|  | GLRLM_SRLGE | -3.330 | <0.001 |
|  | GLRLM_SRHGE | -3.330 | <0.001 |
|  | GLRLM_LRLGE | -3.330 | <0.001 |
|  | GLRLM_LRHGE | -3.389 | <0.001 |
|  | GLRLM_RLNU | -2.093 | 0.036 |
|  | GLRLM_RP | -2.654 | 0.008 |
| **Neighborhood Grey-Level Different Matrix (NGLDM)** | NGLDM_Coarseness | -2.557 | 0.011 |
|  | NGLDM_Contrast | -2.396 | 0.017 |
|  | NGLDM_Busyness | -2.928 | 0.003 |
| **Grey-Level Zone Length Matrix (GLZLM)** | GLZLM_SZE | -3.117 | 0.002 |
|  | GLZLM_LZE | -2.640 | 0.008 |
|  | GLZLM_LGZE | -3.306 | <0.001 |
|  | GLZLM_HGZE | -3.327 | <0.001 |
|  | GLZLM_SZLGE | -2.739 | 0.006 |
|  | GLZLM_SZHGE | -3.348 | <0.001 |
|  | GLZLM_LZLGE | -3.282 | 0.001 |
|  | GLZLM_GLNU | -2.331 | 0.020 |
|  | GLZLM_ZLNU | -3.012 | 0.003 |
|  | GLZLM_ZP | -2.915 | 0.004 |
| **Basic features CT** | HUmax | -3.180 | 0.001 |
|  | CONVENTIONAL_HUQ3 | -2.073 | 0.038 |
|  | HISTO_Kurtosis | -2.196 | 0.028 |
|  | HISTO_ExcessKurtosis | -2.189 | 0.029 |
| **shape** | Volume | -1.987 | 0.047 |
| **Texture Features** | GLRLM_LRHGE | -2.265 | 0.024 |
|  | NGLDM_Contrast | -2.011 | 0.044 |
|  | GLZLM_SZE | -3.131 | 0.002 |
|  | GLZLM_LZHGE | -2.196 | 0.028 |

**Supplemental Table 3.** The correlation between CD8 expression and radiomics features.

| **PET features** | **Features** | ***rho*** | ***p*** |
| --- | --- | --- | --- |
| **Basic features PET** | SUVmin | .296 | .002 |
|  | SUVmean | .268 | .006 |
|  | SUVstd | .269 | .006 |
|  | SUVmax | .273 | .005 |
|  | SUVQ1 | .275 | .005 |
|  | SUVQ2 | .262 | .008 |
|  | SUVQ3 | .262 | .007 |
|  | SUVpeak sphere 0.5mL | .274 | .005 |
|  | SUVpeak sphere 1mL | .254 | .010 |
| **Indices from Histogram** | HISTO_Entropy_log10 | .254 | .010 |
|  | HISTO_Entropy_log2 | .254 | .010 |
|  | HISTO_Energy | -.246 | .012 |
|  | GLCM_Homogeneity | -.275 | .005 |
| **Grey Level Co-occurrence Matrix (GLCM)** | GLCM_Energy | -.227 | .021 |
|  | GLCM_Contrast | .278 | .004 |
|  | GLCM_Entropy_log10 | .220 | .026 |
|  | GLCM_Entropy_log2 | .220 | .026 |
|  | GLCM_Dissimilarity | .275 | .005 |
| **Grey-Level Run Length Matrix (GLRLM)** | GLRLM_SRE | .274 | .005 |
|  | GLRLM_LRE | -.272 | .005 |
|  | GLRLM_LGRE | -.276 | .005 |
|  | GLRLM_HGRE | .266 | .007 |
|  | GLRLM_SRLGE | -.272 | .005 |
|  | GLRLM_SRHGE | .269 | .006 |
|  | GLRLM_LRLGE | -.270 | .006 |
|  | GLRLM_LRHGE | .265 | .007 |
|  | GLRLM_RP | .274 | .005 |
| **Neighborhood Grey-Level Different Matrix (NGLDM)** | NGLDM_Contrast | .310 | .001 |
|  | NGLDM_Busyness | -.247 | .012 |
| **Grey-Level Zone Length Matrix (GLZLM)** | GLZLM_SZE | .297 | .002 |
|  | GLZLM_LZE | -.259 | .008 |
|  | GLZLM_LGZE | -.275 | .005 |
|  | GLZLM_HGZE | .276 | .005 |
|  | GLZLM_SZHGE | .284 | .004 |
|  | GLZLM_LZLGE | -.289 | .003 |
|  | GLZLM_ZLNU | .242 | .014 |
|  | GLZLM_ZP | .283 | .004 |

**Supplemental Table 4.** The correlation between PD-1 expression and radiomics features.

| **PET features** | **Indice** | ***rho*** | ***p*** |
| --- | --- | --- | --- |
| **Basic features** | SUVmin | .320 | .001 |
|  | SUVmean | .315 | .001 |
|  | SUVstd | .307 | .002 |
|  | SUVmax | .309 | .002 |
|  | SUVQ1 | .315 | .001 |
|  | SUVQ2 | .314 | .001 |
|  | SUVQ3 | .309 | .001 |
|  | SUVpeak sphere 0.5mL | .346 | .000 |
|  | SUVpeak sphere 1mL | .356 | .000 |
|  | TLG (mL) | .234 | .018 |
| **Histogram** | HISTO_Entropy_log10 | .313 | .001 |
|  | HISTO_Entropy_log2 | .314 | .001 |
|  | HISTO_Energy | -.310 | .001 |
| **Grey Level Co-occurrence Matrix (GLCM)** | GLCM_Homogeneity | -.258 | .008 |
|  | GLCM_Energy | -.290 | .003 |
|  | GLCM_Contrast | .271 | .006 |
|  | GLCM_Entropy_log10 | .285 | .004 |
|  | GLCM_Entropy_log2 | .285 | .004 |
|  | GLCM_Dissimilarity | .263 | .007 |
| **Grey-Level Run Length Matrix (GLRLM)** | GLRLM_SRE | .226 | .021 |
|  | GLRLM_LRE | -.215 | .029 |
|  | GLRLM_LGRE | -.317 | .001 |
|  | GLRLM_HGRE | .316 | .001 |
|  | GLRLM_SRLGE | -.316 | .001 |
|  | GLRLM_SRHGE | .318 | .001 |
|  | GLRLM_LRLGE | -.310 | .001 |
|  | GLRLM_LRHGE | .314 | .001 |
|  | GLRLM_RLNU | .213 | .031 |
|  | GLRLM_RP | .221 | .025 |
| **Neighborhood Grey-Level Different Matrix (NGLDM)** | NGLDM_Contrast | .271 | .006 |
|  | NGLDM_Busyness | -.231 | .019 |
| **Grey-Level Zone Length Matrix (GLZLM)** | GLZLM_SZE | .278 | .005 |
|  | GLZLM_LZE | -.218 | .027 |
|  | GLZLM_LGZE | -.313 | .001 |
|  | GLZLM_HGZE | .319 | .001 |
|  | GLZLM_SZHGE | .310 | .001 |
|  | GLZLM_LZLGE | -.291 | .003 |
|  | GLZLM_GLNU | .229 | .020 |
|  | GLZLM_ZLNU | .287 | .003 |
|  | GLZLM_ZP | .241 | .014 |
| **Basic features**  **CT features** | HUmean | .200 | .043 |
|  | HUmax | .245 | .013 |
|  | HUQ2 | .215 | .029 |
|  | HUQ3 | .230 | .019 |
|  | HISTO_Skewness | -.194 | .049 |
|  | HISTO_Kurtosis | .213 | .031 |
|  | HISTO_ExcessKurtosis | .213 | .031 |
|  | HISTO_Entropy_log10 | .208 | .035 |
|  | HISTO_Entropy_log2 | .207 | .036 |
|  | HISTO_Energy | -.232 | .018 |
|  | SHAPE_Volume (mL) | .199 | .044 |
|  | SHAPE_Volume (# vx) | .199 | .044 |
|  | SHAPE_Compacity | .226 | .022 |
| **Texture Features** | GLCM_Homogeneity | -.217 | .028 |
|  | GLCM_Energy | -.252 | .010 |
|  | GLCM_Contrast | .280 | .004 |
|  | GLCM_Entropy_log10 | .220 | .026 |
|  | GLCM_Entropy_log2 | .218 | .027 |
|  | GLCM_Dissimilarity | .261 | .008 |
|  | GLRLM_LRHGE | .237 | .016 |
|  | GLRLM_GLNU | .199 | .044 |
|  | GLRLM_RLNU | .206 | .037 |
|  | NGLDM_Contrast | .302 | .002 |
|  | GLZLM_SZE | .254 | .010 |
|  | GLZLM_SZLGE | .226 | .021 |
|  | GLZLM_LZHGE | .214 | .030 |
|  | GLZLM_GLNU | .195 | .049 |
|  | GLZLM_ZLNU | .214 | .030 |
